# Editorial: The Immunomodulatory Roles of Adipocytes

**DOI:** 10.3389/fimmu.2021.827281

**Published:** 2021-12-23

**Authors:** David Bradley, Aimin Xu, Willa A. Hsueh

**Affiliations:** ^1^ Diabetes and Metabolism Research Center, Division of Endocrinology, Diabetes & Metabolism, Department of Internal Medicine, The Ohio State University Wexner Medical Center, Columbus, OH, United States; ^2^ State Key Laboratory of Pharmaceutical Biotechnology, The University of Hong Kong, Hong Kong, Hong Kong SAR, China; ^3^ Department of Medicine, The University of Hong Kong, Hong Kong, Hong Kong SAR, China

**Keywords:** adipocyte, innate and adaptive immune response, exosomes, adipokine cytokines, metabolic disease

Obesity is a global epidemic (1) associated with a state of low-grade, chronic inflammation that enhances the risk of numerous complications, including type 2 diabetes (T2D), non-alcoholic fatty liver disease (NAFLD) and cirrhosis, cardiovascular disease (CVD), cancer, and Alzheimer’s Disease, among others (2–7). A major driver of these conditions is the profound inflammatory changes that occur within the adipose tissue (AT) microenvironment, at the heart of which is the adipocyte. Our understanding of the role of the adipocyte in initiating and propagating innate (Blaszczak et al.) and adaptive (Song and Deng) immune responses in lean and obese states has expanded beyond its classical role in energy storage. The adipocyte produces over 600 cytokines and hormones, collectively called adipokines that modulate chronic inflammation, secretes extracellular matrix proteins that impact metabolism (8, 9), and serves as an immunomodulatory and antigen presenting cell to activate or suppress immune responses within AT and systemically (10). Therefore, the current Research Series “*The Immunomodulatory Roles of the Adipocyte”* highlights a wide range of critical factors originating from the adipocyte that mediate immunity and the metabolic syndrome including extracellular vesicle crosstalk (Huang and Xu), lipid metabolites (Park et al.) and ceramides (Chaurasia et al.), adipocyte fatty acid-binding protein (A-FABP) (Lee et al.), TANK-binding kinase 1 (TBK1) (Zhao and Saltiel), the oncostatin M (Sanchez-Infantes and Stephens), clusterin (Wittwer and Bradley), and leptin (Kiernan and MacIver), illustrating the multi-faceted role of the adipocyte (**Figure 1**). Taken together, this series underscores the much underappreciated role of the adipocyte in the instigation and perpetuation of local and systemic inflammation, leading to the multiple inflammatory-induced complications of obesity.

**Figure 1:**
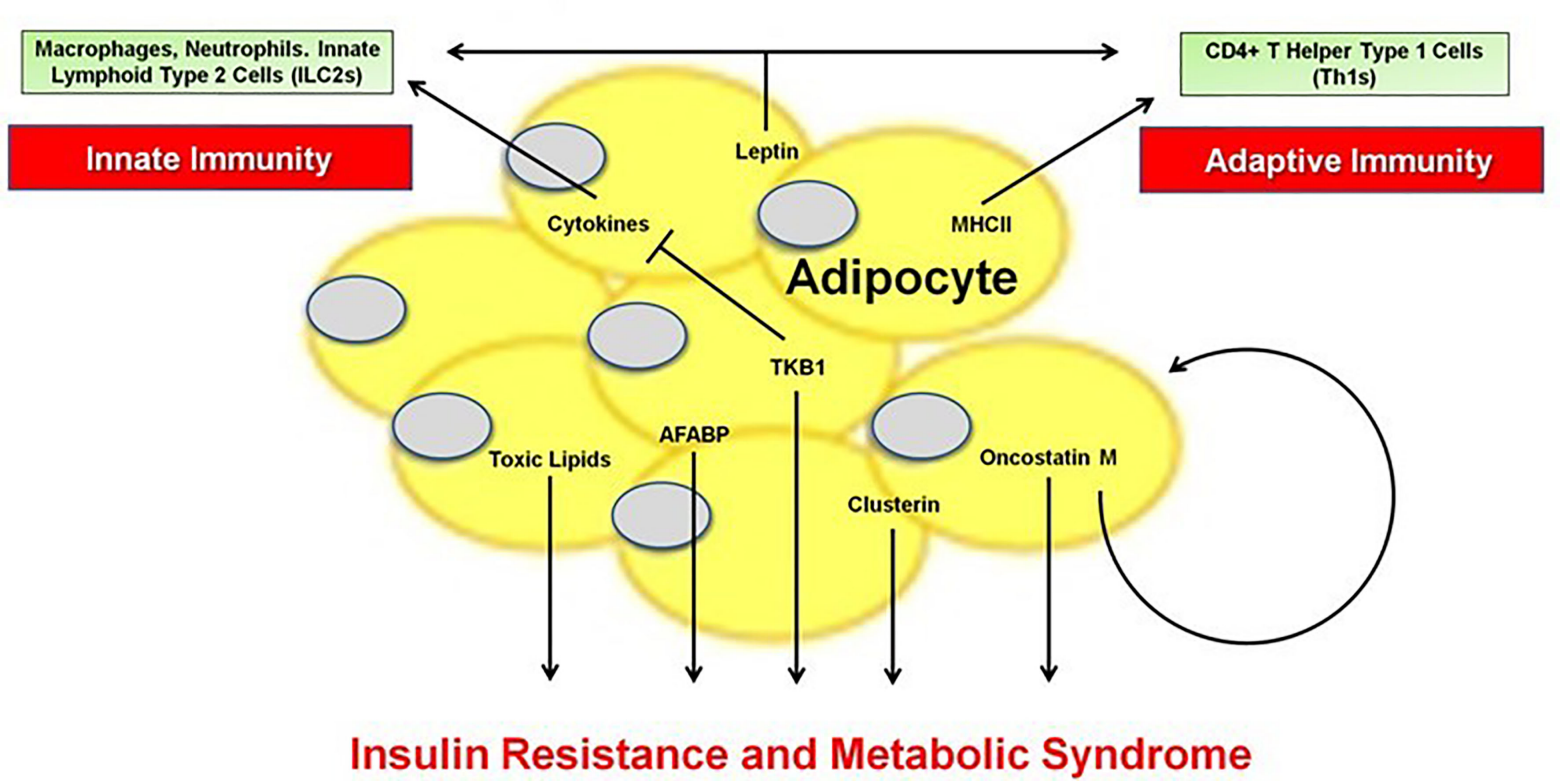
The Innate and Adaptive Immune Functions of the Adipocyte.

An increasingly recognized means of cell-cell communication is through extracellular vesicles (EVs). Huang and Xu nicely summarize the mechanisms by which AT extracellular vesicles (exosomes, microvesicles, and apoptotic bodies) mediate intercellular communications and inter-organ crosstalk, particularly focusing on adipocyte-derived EVs (ADEVs). Exosomes (30-100nm in diameter) arise from multivesicular bodies and are either degraded by the lysosomal pathway or fuse with the plasma membrane and are released from the cell, while microvescicles (MVs, 100-1000 nm is diameter) are pinched off from the plasma membrane and released. EV cargo consists of microRNAs (miRs), mRNAs, proteins, and lipids and are taken up by cells to influence cell development, metabolism, function, and other activities. ADEV production is markedly increased in human and mouse obesity (11). ADEVs impact local immune cells and have been shown to activate AT macrophages (11) and promote monocyte to macrophage conversion (12–14). Adipocytes have been suggested to contribute substantially to miRs in circulating EVs, since adipocyte-specific loss of miR production resulted in a 4-fold drop in EV miR cargo (15). However, it remains unclear how many ADEVs enter the bloodstream (11). Figure 2 of the Huang and Xu review illustrates how ADEVs and their cargo act upon distal organs (including liver, skeletal muscle, pancreas, and brain) to influence the immune system as well as systemic metabolism, while Table 1 summarizes specific functions of various cargo. Finally, this review suggests modified EVs can be used as therapy for metabolic and other diseases.

Activation of a pro-inflammatory pathway leads to the secretion of numerous cytokines (16, 17) that enhance adipocyte lipolysis (18–20), leading to toxic fatty acid species (Chaurasia et al.) and impaired insulin sensitivity (21, 22). Chaurasia et al. review the wide-ranging effects of adipocyte-derived lipotoxicity on inflammation and peripheral tissue dysfunction. Specifically, ceramides, which are sphingolipids located in the cell membrane and within the cell cytosol, associate not only with pro-inflammatory cytokines and circulating free fatty acids, but many obesity-related conditions including insulin resistance, T2D, NAFLD, chronic kidney disease, and adverse CV events including mortality. Inhibition of ceramide synthesis specifically within the adipocyte improves insulin resistance and several of these metabolic derangements in mice, underscoring the role of the adipocyte in providing toxic lipids to incite inflammation (23). Inhibiting ceramide synthesis may be a useful therapeutic strategy for metabolic syndrome. Park et al. further discuss an integrated view of how adipocytes communicate with adipose immune cells using lipid metabolites. Invariant natural killer T (iNKT) cells and γ/δ T cells, rapidly respond to changes in lipid metabolism through sensing lipid antigens loaded on antigen presenting cells (APCs). iNKT cells secrete IL-2, IL-4 and IL-10 which support immunosuppressive regulatory T cells (Tregs), while IL-4 and IL-10 promote anti-inflammatory macrophage M2 polarization (24). However, the lipid antigen is unknown and whether lipid-activated iNKT cells are anti- or proinflammatory remains controversial (25, 26). Similarly, γ/δ T cells are abundantly present in AT and actively interact with adipocytes, but their role in inflammation is also unclear. Nevertheless, iNKT cells and γ/δ T cells are models by which adipocytes can present a lipid antigen to activate an immune cell.

Song and Deng further define the adipocyte as a novel APC, substantially contributing to adaptive immunity in AT. The adipocyte major histocompatibility II (MHCII) pathway is markedly enhanced in obesity during which it is stimulated primarily by interferon-γ (IFNγ) (10, 27). Adipocyte antigen presentation to naïve T cells promotes inflammatory Th1 effector cell activation, while further production of IFNγ fosters more adipocyte MHCII production, resulting in an escalating cycle of AT inflammation. Mice with genetic depletion of adipocyte MHCII, gain the same amount of weight as control mice, but are protected from AT inflammation and insulin resistance. Within obese AT, adipocytes also activate innate immune cells including macrophages and neutrophils to promote inflammation, while innate lymphoid cells type 2 may be metabolically protective, as reviewed by Blaszczak et al. They highlight the central role of the adipocyte in linking the innate and adaptive immune systems through the secretion of adipokines and cytokines; exosome release of lipids, hormones, and microRNAs; and contact interaction with other immune cells. During diet-induced obesity, a negative feedback loop involving the non-canonical IKK family member TBK1 regulates both innate immunity and glucose and energy metabolism within the adipocyte, as reviewed by Zhao and Saltiel. Upon activation by inflammatory cytokines and lipids, TBK1 suppresses NFκB signaling and attenuates AMP kinase-mediated metabolic activity. They suggest the potential of a TBK1/IKK inhibitor as a new therapy for metabolic diseases.

Adipocytes secrete a multitude of factors that have either pro- or anti-inflammatory functions that impact systemic metabolism. Leptin, one of the most well-known hormones secreted by adipocytes in obesity, in addition to its metabolic function, has important pro-inflammatory actions as comprehensively summarized by Kiernan and MacIver. They provide data suggesting that nearly every immune cell is activated by leptin. Oncostatin M (OSM) is a proinflammatory cytokine, elevated in human obesity and metabolic disease, which inhibits preadipocyte differentiation and enhances the proinflammatory response of adipocytes in a paracrine manner (Sanchez-Infantes and Stephens). However, loss of this system by genetic ablation of the OSM receptor in adipocytes, in contrast to these findings, also aggravates glucose homeostasis, so Sanchez-Infantes and Stephens argue that some adipocyte inflammation is necessary for normal metabolic function. Lee et al. focus on A-FABP, a lipid chaperone abundantly secreted from adipocytes and macrophages, as a key player mediating adipose-vascular cross-talk. A-FABP, in part *via* its activation of c-Jun NH2-terminal kinase (JNK) and activator protein-1 (AP-1), forms a positive feedback loop to perpetuate inflammatory responses. In mice, selective JNK inactivation in the AT significantly reduced expression of A-FABP and circulating A-FABP levels and alleviated high fat high cholesterol diet-induced atherosclerosis (28). In humans, raised circulating AFABP levels are associated with incident metabolic syndrome, T2D and CVD, as well as nonalcoholic steatohepatitis, diabetic nephropathy and adverse renal outcomes, all conditions closely related to inflammation and enhanced CV mortality (Lee et al.; 29–34). They suggest that A-FABP may be a therapeutic target in obesity-related complications. Finally, various extracellular matrix proteins (ECM) are secreted by adipocytes, which in turn, determines the AT architecture, enhances inflammation, and regulates systemic metabolism. As discussed by Wittwer and Bradley (8), adipocyte ECM production is amplified in obesity, resulting in AT fibrosis and adipocyte hypoxia. Clusterin (apolipoprotein J), an ECM-related protein whose expression and secretion in adipocytes is higher in human obesity, is associated with multiple metabolic syndrome components and CV risk and has key effects centrally to modulate amyloid-beta in Alzheimer’s Disease. The insulin antagonizing effects of clusterin appear to be in the liver (8).

In summary, the adipocyte exerts immunomodulatory functions *via* multiple novel mechanisms to regulate inflammation and contribute to obesity-related disease. The original research articles and review papers included in this issue present a range of topics under active investigation. Understanding this function and how it impacts other AT immune cells and obesity-related complications is critical to prevention and treatment. Yet, despite a recognition of the importance of adipocytes in inflammatory dysregulation, the mechanisms underlying the inflammatory regulation of these disorders are not fully understood and should remain a critical focus for future investigation.

## Author Contributions

All authors listed have made a substantial, direct and intellectual contribution to the work, and approved it for publication.

## Funding

This study was supported by grants from the American Diabetes Association 1-16-ICTS-049, The National Institutes of Health KL2 Scholar Award KL2TR001068 and HL135622.

## Conflict of Interest

The authors declare that the research was conducted in the absence of any commercial or financial relationships that could be construed as a potential conflict of interest.

## Publisher’s Note

All claims expressed in this article are solely those of the authors and do not necessarily represent those of their affiliated organizations, or those of the publisher, the editors and the reviewers. Any product that may be evaluated in this article, or claim that may be made by its manufacturer, is not guaranteed or endorsed by the publisher.
